# Cardiac length-dependent activation driven by force-dependent thick-filament dynamics

**DOI:** 10.1016/j.bpj.2024.05.025

**Published:** 2024-05-28

**Authors:** Alexandre Lewalle, Gregory Milburn, Kenneth S. Campbell, Steven A. Niederer

**Affiliations:** 1 National Heart and Lung Institute, Faculty of Medicine, Imperial College London, London, United Kingdom; 2 Department of Physiology, University of Kentucky, Lexington, Kentucky; 3 Division of Cardiovascular Medicine, University of Kentucky, Lexington, Kentucky

## Abstract

The length-dependent activation (LDA) of maximum force and calcium sensitivity are established features of cardiac muscle contraction but the dominant underlying mechanisms remain to be fully clarified. Alongside the well-documented regulation of contraction via the thin filaments, experiments have identified an additional force-dependent thick-filament activation, whereby myosin heads parked in a so-called off state become available to generate force. This process produces a feedback effect that may potentially drive LDA. Using biomechanical modeling of a human left-ventricular myocyte, this study investigates the extent to which the off-state dynamics could, by itself, plausibly account for LDA, depending on the specific mathematical formulation of the feedback. We hypothesized four different models of the off-state regulatory feedback based on (A) total force, (B) active force, (C) sarcomere strain, and (D) passive force. We tested if these models could reproduce the isometric steady-state and dynamic LDA features predicted by an earlier published model of a human left-ventricle myocyte featuring purely phenomenological length dependences. The results suggest that only total-force feedback (A) is capable of reproducing the expected behaviors, but that passive tension could provide a length-dependent signal on which to initiate the feedback. Furthermore, by attributing LDA to off-state dynamics, our proposed model also qualitatively reproduces experimentally observed effects of the off-state-stabilizing drug mavacamten. Taken together, these results support off-state dynamics as a plausible primary mechanism underlying LDA.

## Significance

The mechanistic origins of length-dependent activation (LDA), a fundamental regulatory feature of cardiac muscle contraction, are still debated. Growing experimental evidence suggests that myosin cross-bridges transition between a tension-generating “on” state and an inactive “off” state in a tension-dependent manner. Using a minimal biophysical model, we tested the hypothesis that the resulting feedback effect could potentially constitute a major contribution to LDA. By assuming a force-dependent feedback, the model quantitatively reproduces the key features of LDA. It also reproduces qualitatively the main observed effects of the off-state-targeting drug mavacamten. This provides a modeling framework for investigating the impact of off-state dynamics on whole-heart physiology and its clinical implications.

## Introduction

Muscle sarcomeres contract when myosin heads in the thick filaments bind and pull on actin thin filaments (1). The tuning of the contraction strength by sarcomere stretch (length-dependent activation, LDA) (2) has been presented as the microscopic underpinning of the Frank-Starling effect, whereby, in the macroscopic muscle system, the strength of contraction increases with tissue stretch (3,4,5). At the cellular level, LDA manifests itself primarily as length dependences in 1) the maximum active tension at high $\left[{\text{Ca}}^{2+}\right]$ and 2) the sensitivity of active tension to changes in $\left[{\text{Ca}}^{2+}\right]$ (6). An early explanation of length-dependent tension in the short sarcomere described the thin filaments interfering sterically with each other across the M line, and the thick filament “crumpling or folding” against the Z discs, resulting in a decreasing active tension with shortening (7). However, LDA is arguably the cumulative outcome of multiple alternative submechanisms acting in parallel (8): changes in filament proximity (9,10), TnC affinity for ${\text{Ca}}^{2+}$ (11,12,13), thin-filament cooperativity (14,15), mechanisms associated with myosin-binding protein C (MyBP-C) (16,17), and interfilament electrostatics (18). The relative importance of these contributions to tension regulation may vary according to muscle type and species, which makes a systematic quantitative characterization of LDA and of its role in whole-heart function challenging.

There is presently growing evidence that thick-filament activation could also contribute to LDA. This effect appears to be itself tension-dependent (19,20,21). It correlates with a structural transformation of the thick-filament structure from a “parked” OFF state, where the myosin heads are folded against the filament core (22,23), to an ON state where the head orientations allow interaction with the actin thin filaments (24,25,26,27). A mechanosensitive positive feedback effect emerges whereby sarcomere tension releases myosin heads from the OFF to ON state to generate further tension. However, the detailed nature of this feedback and the precise extent of its contribution remain to be established. For instance, some authors (28) reported a dependence of LDA on the isoform of titin, the sarcomere component associated with passive stress (29). In contrast, others have reported the thick filaments being in the OFF configuration regardless of the diastolic sarcomere length, and therefore of passive tension (30). Arguably, the ambiguous attribution of the feedback to length or tension stems from the interrelationship between strain and stress within sarcomeres. The aim of this study was to use biophysical modeling as a tool for assessing the inherent ability of different hypothetical feedback formulations to account for emergent length-dependent behavior. Clarifying the mechanisms of OFF-state behavior is becoming all the more relevant, given the established clinical importance of controlling OFF-state dynamics pharmacologically, for example, with the drug mavacamten (31,32,33,34).

Long before the discovery of the OFF state, some biomechanical models of myocyte contraction were implementing LDA by phenomenologically inserting length-dependent terms in, e.g., the tension magnitude, $\left[{\text{Ca}}^{2+}\right]$ sensitivities, or kinetic rate constants (35,36,37,38,39). This ad hoc approach at best provides an empirical catch-all description of multiple biophysical mechanisms that limits a systematic consideration of specific mechanisms. More recent modeling studies have demonstrated the ability of OFF-state dynamics to predict observed LDA behavior by assuming a purely force-dependent (passive + active) formulation of the OFF-state feedback (40,41). Given the strong interconnectedness between stress and strain, alternative formulations cannot be dismissed a priori. However, to our knowledge, a critical comparison of potential formulations has not been reported to date. We therefore tested different feedback paradigms, where the myosin OFF/ON state transitions are governed explicitly by (A) the total force $F_{\text{total}}$, (B) the active force $F_{\text{active}}$, (C) the sarcomere strain *C*, or (D) the passive force $F_{\text{passive}}$. By computationally simulating these paradigms, we sought to test whether the myosin OFF state could plausibly account for length-dependent sarcomere properties without resorting to ad hoc phenomenological formulations of LDA. In other words, can we identify, on the basis of a formal mathematical description of the OFF state, an upper limit to the proportion of LDA that can be attributed to OFF-state dynamics alone?

We amended the 2017 human ventricular contraction model of Land et al. (39) by introducing an OFF-state subsystem. In its original formulation, the Land model implements LDA by imposing explicit ad hoc length-dependent terms on the tension magnitude (parameter $T_{\text{ref}}$) and on the calcium sensitivity of troponin (Ca_T50_). Using a graphical approach, we tested each of the above OFF-state regulation paradigms for their inherent ability to reproduce the original model’s predictions of LDA, as manifested in the isometric steady-state force-calcium relationship (F-pCa). The test criterion was the ability to simultaneously reproduce the length dependences of the maximum force ($F_{\max}$) and the calcium sensitivity (${\text{pCa}}_{50}$). For this purpose, it suffices to emulate the existing calibrated model, instead of fitting the test models afresh from new experimental data. This also allows the role of passive tension to be investigated through the variation of the passive sarcomere stiffness.

Having identified a plausible mechanism, we tested its effectiveness at predicting experimental observations of the effect of mavacamten, a drug known to stabilize the OFF state (42,43). In particular we considered the F-pCa and dynamic stiffness measurements of Awinda et al. (44) and Ma et al. (45). To provide a benchmark, we reparameterized the model to reproduce the main features of the Awinda control measurements before then simulating mavacamten. Overall, our results qualitatively reproduce the essential experimental observations, supporting the plausibility of myosin OFF-state dynamics as a potentially dominant contributor to sarcomere tension regulation and LDA.

For decades, biophysical models have played a valuable role in providing a theoretical framework for integrating experimental measurements and observations into a unified picture, and also for guiding further experimental investigation through computational simulations. Recent years have seen a growing ambition to exploit such models as tools to aid medical diagnosis and interventions and in the development of targeted pharmacological treatments (46,47). The relevance of the myosin OFF state is becoming increasingly recognized, in particular in the context of treating hypertrophic cardiomyopathy (33,48). Within this perspective, our theoretical formalism proposes a mechanistic framework for further investigating this key phenomenon.

## Methods

### Model formulation

**Figure 1:**
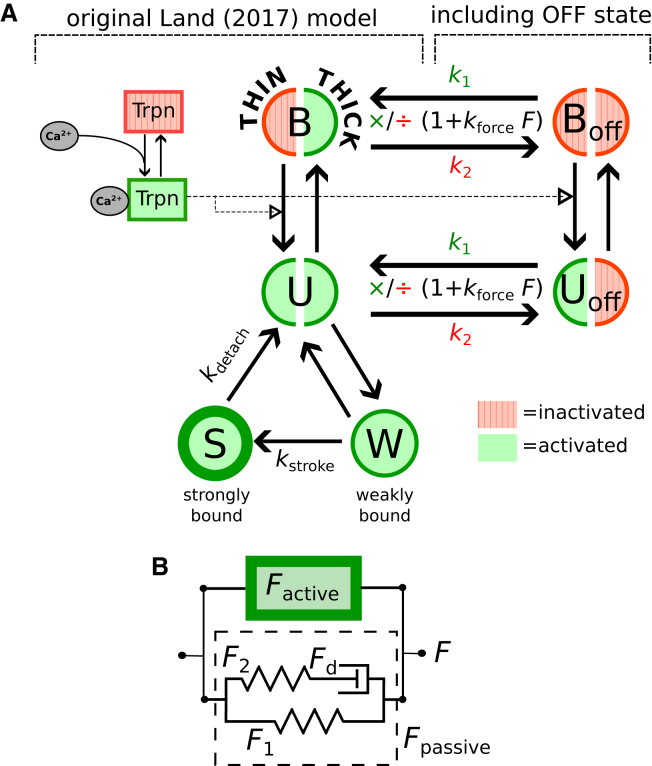
Contraction model of Land et al., amended to include OFF-state dynamics. (*a*) Kinetic scheme, featuring the states in the original model (39): *B*, thin-filament binding sites blocked by tropomyosin; *U*, unblocked; *W*, thin filaments with weakly bound cross-bridges; and *S*, strongly bound. Each system state comprises two substates associated with the thin-filament (*left semicircle*) and thick-filament (*right semicircle*) activation status. The absence or presence of thin-thick filament binding interaction, for each state symbol, is represented pictorially by means of a gap separating the left and right semicircles. The additional states, representing myosin heads in the OFF state, are $B_{\text{off}}$ and $U_{\text{off}}$, which have the actin binding blocked or unblocked by tropomyosin, respectively, mirroring *B* and *U*. Force dependence in the OFF-state dynamics is implemented by either multiplying the OFF-to-ON rate constant $k_{1}$—or dividing the ON-to-OFF rate constant $k_{2}$—by $\left(1+k_{\text{force}}F_{\text{total}}\right)$ (see Eqs. 5 and 6). (*b*) Schematic representation of the total sarcomere force $F_{\text{total}}=F_{\text{active}}+F_{\text{passive}}$, with $F_{\text{active}}$ calculated from the kinetic model using Eq. 2 and $F_{\text{passive}}$ modeled as a simple spring-dashpot system with nonlinear elastic elements (39). To see this figure in color, go online.

The Land et al. (39) contraction model describes the kinetics of cross-bridge and actin interactions from a mass-action-like perspective, represented by a system of ordinary differential equations (ODEs). Each state in the model (*left side* of Fig. 1 *a*) may be visualized as a combination of thin- and thick-filament states. In the original model formulation, the thin filaments are regulated through their ${\text{Ca}}^{2+}$ dynamics; there is no regulatory mechanism acting specifically on the thick filaments. In summary, the states *B* and *U* represent thin-filament configurations with actin binding sites, respectively, blocked and unblocked by tropomyosin. In both these states, myosin heads are unbound from actin. Actin binding occurs starting from state *U*, leading to the tension-bearing states *W* (weakly bound, “prestroke”) and *S* (strongly bound, “poststroke”). The power stroke occurs during the transition from *W* to *S*. The net average cross-bridge force is hence given by the population of the post-stroke state *S* as well as by the time-dependent distortions ($\zeta_{S}$ and $\zeta_{W}$) of the *S* and *W* states, both of which count as additional state variables in the system (see appendix). The kinetics of this ODE system is governed by: the transition rates between states *U* and *W* and between *W* and *S*; the strain-dependent detachment rate constants from states *W* and *S*; and the decay rate of cross-bridge deformations in the *W* and *S* states.

We amended the above framework to include the myosin OFF/ON dynamics by introducing two new states: $B_{\text{off}}$ and $U_{\text{off}}$ (*right side* of Fig. 1 *a*). These states respectively mirror *B* and *U* but with the myosin switched OFF. This scheme accounts for the four possible combinations of the thin- and thick-filament configurations before binding (i.e., exposed/nonexposed and ON/OFF). As in the original model, cross-bridge binding to actin occurs only during the transition U→W when, simultaneously, the thin-filament sites are exposed and the myosin heads are switched ON. The binding of the myosin heads to actin is represented pictorially in Fig. 1 *a* by the joining of the left and right semicircles in the state symbols. The transition $B_{\text{off}}↔U_{\text{off}}$ and B↔U thus have identical rate constants, as they represent thin-filament transitions that occur independently of the thick filaments. Likewise, $B↔B_{\text{off}}$ and $U↔U_{\text{off}}$ represent identical thick-filament transitions, parameterized with the rate constants $k_{1}$ and $k_{2}$.

The Land model describes the total force exerted by the sarcomere as the sum of active and passive contributions: $F_{\text{total}}=F_{\text{active}}+F_{\text{passive}}$, where $F_{\text{passive}}$ is a three-component mechanical system (Fig. 1 *b*), which, in the isometric steady state, is determined by the parallel elastic component:

(Equation 1) $$F_{\text{passive}}=F_{1}=a\left(e^{bC}-1\right)$$

where *C* denotes the strain and the constants *a* and *b* define nonlinear stiffness characteristics. The dashpot is formulated as a linear damper, albeit with different viscosities depending on the direction of change in the dashpot strain $C_{d}$, i.e., the tension across the dashpot is $F_{d}=a\eta_{\pm }\cdot dC_{d}/dt$, with ± denoting the sign of $dC_{d}/dt$. This formalism was unaltered in this study.

**Figure 2:**
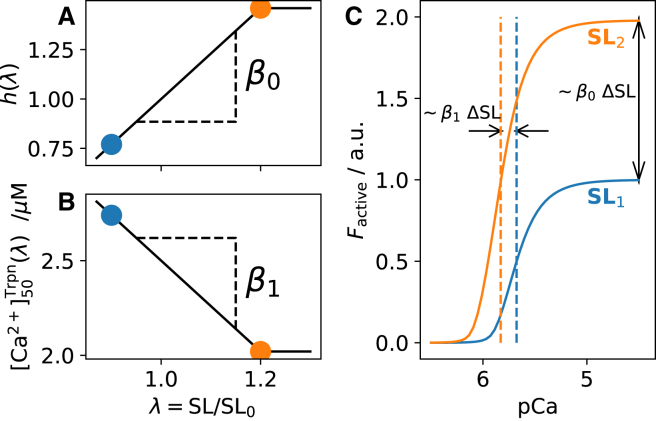
Phenomenological length dependence of sarcomere mechanics, as implemented in the original Land model. (*a*) The overall force magnitude is multiplied, ad hoc, by the phenomenological function $h\left(\lambda ;\beta_{0}\right)$, where $\lambda =\text{SL}/{\text{SL}}_{0}$ is the extension factor (${\text{SL}}_{0}$ is the resting sarcomere length) (39). In the range 0.87<λ<1.2, h(λ) varies linearly with gradient $\beta_{0}$. (*b*) The calcium sensitivity ${\left[{\text{Ca}}^{2+}\right]}_{50}$ (Eq. 4) is also assumed to vary linearly with a gradient $\beta_{1}$, for λ<1.2. (*c*) The effect of $\beta_{0}$ and $\beta_{1}$ manifests itself in the characteristic features of the F-pCa relationship (i.e., the maximum force and the pCa of the half-maximum point). This work seeks to reproduce these effects by means of the OFF-state dynamics instead of relying on $\beta_{0}$ and $\beta_{1}$. To see this figure in color, go online.

Following the Land model, we describe the active tension as the sum of contributions from the cross-bridge strains $\zeta_{S}$ and $\zeta_{W}$ of the system while it is in the *W* and *S* states, respectively. In the original model,

(Equation 2) $$F_{\text{active}}=h\left(\lambda \right)\frac{T_{\text{ref}}}{r_{S}}\left[\left(\zeta_{S}+1\right)S+\zeta_{W}W\right]$$

where $r_{S}$ denotes the steady-state duty ratio for state *S* and $T_{\text{ref}}$ (kPa) is a scaling factor. The function

(Equation 3) $$h\left(\lambda \right)=1+\beta_{0}\left(\lambda -1\right)$$

modulates the tension magnitude as an ad hoc sarcomere-length (SL) dependence, with $\lambda =C+\;1\;=\;\text{SL}/{\text{SL}}_{0}$ being the ratio of the sarcomere length SL to the resting length SL_0_ (Fig. 2 *a*). The presently amended model discards this ad hoc contribution by setting $\beta_{0}=0$, so that any SL dependence emerges instead naturally from the OFF-state dynamics.

The Land model further postulates an ad hoc SL-dependent $\left[{\text{Ca}}^{2+}\right]$ sensitivity via the half-activation concentration for troponin:

(Equation 4) $${\left[{\text{Ca}}^{2+}\right]}_{50}^{\text{Trpn}}\left(\lambda \right)={\left[{\text{Ca}}^{2+}\right]}_{50}^{\text{Trpn},\text{ref}}+\beta_{1}\left(\lambda -1\right)$$

where ${\left[{\text{Ca}}^{2+}\right]}_{50}^{\text{Trpn},\text{ref}}$ and $\beta_{1}$ (<0) are fixed parameters. This ad hoc dependence is also discarded in the new framework by setting $\beta_{1}=0$. In terms of the F-pCa relationship, $\beta_{0}$ and $\beta_{1}$ give rise, respectively, to the SL dependences of the maximum saturating force $F_{\max}$ and the net $\left[{\text{Ca}}^{2+}\right]$ sensitivity (${\text{pCa}}_{50}$, the value of pCa at half-activation), as illustrated in Fig. 2 *c*.

The amended framework replaces the ad hoc terms $\beta_{0}$ and $\beta_{1}$ with force-dependent dynamics in the OFF state, implemented via $k_{1}$ and $k_{2}$. Given the experimental evidence that the transition between the OFF and ON states is mechanosensitive, some previous modeling studies have associated OFF-state dynamics with a force-dependent enhancement of $k_{1}$ in the following form (40,41):

(Equation 5) $$\left\{ \begin{array}{cc} k_{1}=k_{1}^{0}\times \left(1+k_{\text{force}}F_{\text{total}}\right) & \\ k_{2}=k_{2}^{0} & \end{array}\right.$$

where $k_{1}^{0}$ and $k_{2}^{0}$ denote the baseline values in the absence of force, $F_{\text{total}}$ is the total tension in the sarcomere, and $k_{\text{force}}$ is a constant. In principle, one may also consider an alternative hypothetical scenario where, instead, $k_{2}$ is suppressed:

(Equation 6) $$\left\{ \begin{array}{c} k_{1}=k_{1}^{0}\\ k_{2}=k_{2}^{0}÷\left(1+k_{\text{force}}F_{\text{total}}\right) \end{array}\right.$$

In either case, the isometric steady-state balance between the ON and OFF states is governed not by the absolute values of $k_{1}^{0}$ and $k_{2}^{0}$ but by the equilibrium constant

(Equation 7) $$K_{\text{OFF}}=\frac{k_{1}^{0}}{k_{2}^{0}}\times \left(1+k_{\text{force}}F_{\text{total}}\right)$$

**Figure 3:**
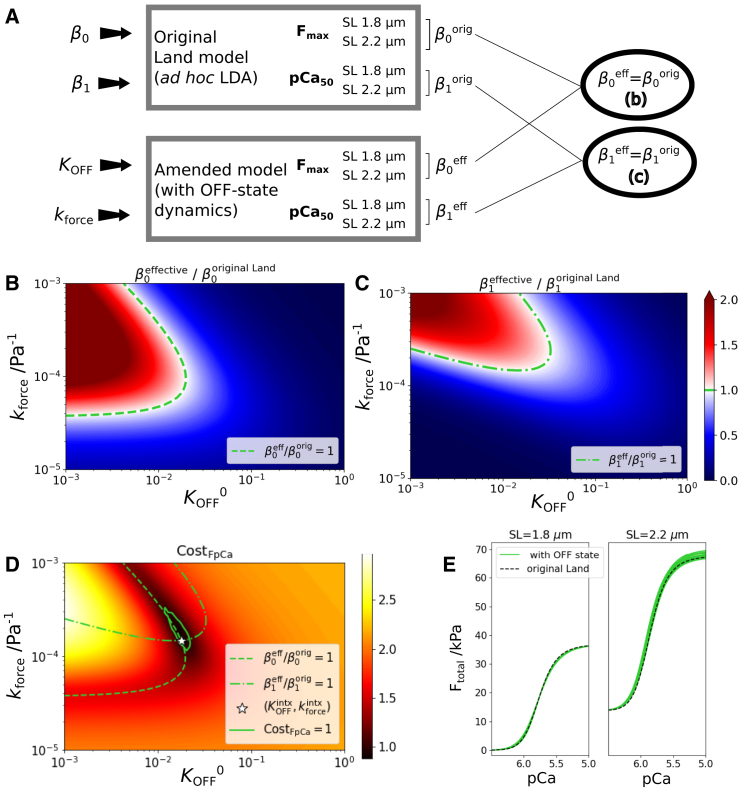
Matching isometric steady-state SL dependences of the OFF-state-based model and the original Land model. (*a*) Schematic explanation of the analysis. F-pCa curves are simulated for SL = 1.8 and 2.2 μm using the original Land model (39) and the amended model assuming test values $K_{\text{OFF}}^{0}$ and $k_{\text{force}}$. Consistency between the two models is assessed by determining the effective values $\beta_{0}^{\text{effective}}$ and $\beta_{1}^{\text{effective}}$ for the test model and comparing them with the $\beta_{0}$ and $\beta_{1}$ of the original model. The ratios (*b*) $\beta_{0}^{\text{effective}}/\beta_{0}^{\text{original}}$ and (*c*) $\beta_{1}^{\text{effective}}/\beta_{1}^{\text{original}}$ are mapped out over the space of $K_{\text{OFF}}^{0}$ and $k_{\text{force}}$. The green contours in (*b*) and (*c*) correspond to value 1, and hence represent the subset of test models (i.e., $K_{\text{OFF}}^{0}$ and $k_{\text{force}}$ values) that correctly emulate the original model at the two SLs considered. (*d*) The intersection of these contours at ($K_{\text{OFF}}^{\text{intx}}$, $k_{\text{force}}^{\text{intx}}$) identifies the test parameters that simultaneously match $\beta_{0}$ and $\beta_{1}$. The cost function, mapped out over the parameter space around ($K_{\text{OFF}}^{\text{intx}}$, $k_{\text{force}}^{\text{intx}}$), identifies the “region of consistency” where the two models agree to within errors $\sigma_{F\max}∼3\;\text{kPa}$ and $\sigma_{{\text{Ca}}_{50}^{2}}∼0.2\;\mu M$. (*e*) This consistency is demonstrated by simulating the F-pCa curves for test values of $K_{\text{OFF}}^{0}$ and $k_{\text{force}}$ sampled within the consistency region (*green curves*) and comparing them with the original Land model predictions (*dashed black curves*). To see this figure in color, go online.

To test the hypotheses that LDA behavior can be plausibly reproduced by replacing the ad hoc parameters $\beta_{0}$ and $\beta_{1}$ with an OFF-state model, we considered the following variants to Eq. 7:

(Equation 8) $$\text{Paradigm}\;A:K_{\text{OFF}}=K_{\text{OFF}}^{0}\times \left(1+k_{\text{force}}F_{\text{total}}\right)$$

(Equation 9) $$\text{Paradigm}\;B:K_{\text{OFF}}=K_{\text{OFF}}^{0}\times \left(1+k_{\text{active}}F_{\text{active}}\right)$$

(Equation 10) $$\text{Paradigm}\;C:K_{\text{OFF}}=K_{\text{OFF}}^{0}\times \left(1+k_{C}C\right)$$

(Equation 11) $$\text{Paradigm}\;D:K_{\text{OFF}}=K_{\text{OFF}}^{0}\times \left(1+k_{\text{passive}}F_{\text{passive}}\right)$$

where $K_{\text{OFF}}^{0}=k_{1}^{0}/k_{2}^{0}$ is the baseline equilibrium constant, C=λ−1 is the sarcomere strain (39), and $k_{\text{force}}$, $k_{\text{active}}$, $k_{C}$, and $k_{\text{passive}}$ represent the feedback gain (generically $k_{\text{feedback}}$). Our general approach is schematized in Fig. 3 *a*. The amended model is defined by setting $\beta_{0}=\beta_{1}=0$ and replacing these ad hoc SL dependences with the states $B_{\text{off}}$ and $U_{\text{off}}$. We then look for $K_{\text{OFF}}$ and $k_{\text{force}}$ values that, for the chosen feedback paradigm (Eqs. (8), (9), (10) and (11)), match the LDA predictions of the original model. A match in the predictions therefore signifies that the SL dependences of $F_{\max}$ and $k_{\text{force}}$ arise exclusively from the OFF-state subsystem.

### Experiment simulations

The state-transition kinetics of the original Land model is defined by a set of seven ODEs. To implement the OFF-state dynamics, we introduce two additional ODEs associated with $B_{\text{off}}$ and $U_{\text{off}}$ (see appendix). Experiments were simulated by solving this ODE system after applying appropriate initial conditions and constraints:

*F-pCa simulations* describe the relationship between force and $\left[{\text{Ca}}^{2+}\right]$ in the isometric steady state. Under this condition, all the ODE time derivatives are set to zero and exact solutions for the state populations can be determined in closed form (see appendix). For a given externally imposed sarcomere length SL, the model returns a unique F-pCa relationship, which is conventionally fitted to a Hill-type function:

(Equation 12) $$F_{\text{total}}\left(\text{pCa};\text{SL}\right)=F_{\text{passive}}\left(\text{SL}\right)+\frac{F_{\max}}{1+10^{n_{H}\left(\text{pCa}-{\text{pCa}}_{50}\right)}}$$

The curve parameters $F_{\max}$, $n_{H}$, and ${\text{pCa}}_{50}$ were determined by fitting the simulated curves using a Nelder-Mead algorithm implemented with the minimize function in the scipy.optimize library.

*Dynamic stiffness measurements* were simulated by applying a small-amplitude (0.1%) sinusoidal variation $\Delta \lambda_{\sin}$ of a given frequency *f* to the SL and allowing the system to reach its limit cycle (typically after ∼10 oscillations). The model responds to an externally applied length variation λ(t) via the passive element (C=λ−1 in Eq. 1) and also the cross-bridge distortions $\zeta_{W}$ and $\zeta_{S}$ (Eq. 16 in the appendix). The time-dependent ODE solutions were obtained using the LSODA solver implemented in scipy.integrate.odeint. The resulting force during recovery, adapted from Eq. 2, was hence given by

(Equation 13) $$F_{\text{total}}\left(t;\text{SL}\right)=F_{\text{passive}}\left(\text{SL}\right)+F_{\text{active}}\left(t;\text{SL}\right)$$

The dynamic (complex) stiffness was determined at that frequency by dividing the sinusoidal force response perturbation by the applied strain: $E\left(f\right)=\Delta F_{\sin}\left(f\right)/\Delta \lambda_{\text{si}}$.

*Mavacamten* stabilizes the OFF state, consistent with a reduction of the equilibrium constant $K_{\text{OFF}}$ (Eq. 7). This was achieved within the above modeling framework by amending Eq. 5 as

(Equation 14) $$\left\{ \begin{array}{c} k_{1}^{\text{Mav}}=r\times k_{1}^{0}\times \left(1+k_{\text{force}}F_{\text{total}}\right)\\ k_{2}^{\text{Mav}}=k_{2}^{0} \end{array}\right.$$

with 0<r<1. We determined the value of *r* that reduces $F_{\max}$ by 30%, a typical value reported in several recent mavacamten experiments (44,45). F-pCa and dynamic stiffness measurement simulations were then performed as explained above. We note in passing that consistency with a reduced $K_{\text{OFF}}$ is also achievable in principle by, e.g., dividing $k_{2}^{0}$ by *r*, or multiplying 1 or $k_{\text{force}}$ by *r*, or indeed any combination of these modifications. Having explored these alternative formulations, we found no significant systematic impact in the comparisons with experiments reported in the results.

### Testing the hypotheses

Our purpose is to test the hypotheses that the LDA properties of the Land model, manifested by the length dependences of $F_{\max}$ and ${\text{pCa}}_{50}$ in the F-pCa curves, can arise purely from either of the OFF-state feedback subsystems proposed in Eqs. 8, 9, 10, and 11.

In general, a given combination of $K_{\text{OFF}}^{0}$ and $k_{\text{feedback}}$ (=$k_{\text{force}}$ for paradigm A, $k_{\text{active}}$ for paradigm B, $k_{C}$ for paradigm C, or $k_{\text{passive}}$ for paradigm D) potentially affects both the absolute and relative changes in $F_{\max}$ and ${\text{pCa}}_{50}$ with varying length. To resolve these length dependences at two stretch values (i.e., four observables) it is therefore necessary to modify two additional model parameters, e.g., $T_{\text{ref}}$ and ${\left[{\text{Ca}}^{2+}\right]}_{50}^{\text{Trpn},\text{ref}}$ (the main determinants of $F_{\max}$ and ${\text{pCa}}_{50}$ in the original model). Thus, we tested each feedback paradigm in turn by setting $\beta_{0}=\beta_{1}=0$ and then fitting $K_{\text{OFF}}^{0}$, $k_{\text{feedback}}$, $T_{\text{ref}}$, and ${\left[{\text{Ca}}^{2+}\right]}_{50}^{\text{Trpn},\text{ref}}$ to match the target values of $F_{\max}$ and ${\text{pCa}}_{50}$ predicted by the Land model for stretches λ=1.0 and λ=1.2. The solution can therefore be understood as producing effective $\beta_{0}$ and $\beta_{1}$ values (between the two chosen stretch points) that are equivalent to the ad hoc parameters in the original model.

The unique determination of the above parameters, within a given feedback paradigm, relies on the existence of a solution in the first place. Considering that $K_{\text{OFF}}^{0}$ and $k_{\text{feedback}}$ are the primary parameters of interest describing the OFF-state dynamics, we assessed this fitting ability by drawing a map of $K_{\text{OFF}}^{0}$ versus $k_{\text{feedback}}$, and identifying the sets of points where the effective $\beta_{0}$ and $\beta_{1}$ values matched the target values of the original model. Their intersection therefore identifies the parameter pair ($K_{\text{OFF}}^{0}$, $k_{\text{feedback}}$) that achieves both targets simultaneously.

## Results

### Matching F-pCa length dependences

Here, we illustrate the analysis approach for paradigm A (i.e., feedback on total force). Results for paradigms B–D, summarized in the next section, were obtained by a straightforward adaptation.

Effective values $\beta_{0}^{\text{effective}}$ and ${\beta_{1}}^{\text{effective}}$ were computed over a range of the parameter space defined by $K_{\text{OFF}}^{0}$ and $k_{\text{force}}$. Color maps of the ratios $\beta_{0}^{\text{effective}}/\beta_{0}^{\text{Land}}$ and $\beta_{1}^{\text{effective}}/\beta_{1}^{\text{Land}}$ are shown in Fig. 3, *b* and *c*, respectively. In each case, the green curves represent the contours of value 1, identifying the locus of points that satisfy the matching of the predicted $F_{\max}$ and ${\text{pCa}}_{50}$ individually. These contours are combined in Fig. 3 *d*, and their intersection point $\left(K_{\text{OFF}}^{\text{intx}}=0.0158,k_{\text{force}}^{\text{intx}}=1.48\times 10^{-4}\;{\text{Pa}}^{-1}\right)$ therefore represents the optimal matching of the models with respect to $F_{\max}$ and ${\left[{\text{Ca}}^{2+}\right]}_{50}$ simultaneously. For this test model, the recalibrated Land parameters were $T_{\text{ref}}=109\;\text{kPa}$ and ${\left[{\text{Ca}}^{2+}\right]}_{50}^{\text{Trpn},\text{ref}}=1.17\;\mu M$.

To appraise the strength of the constraints of $K_{\text{OFF}}^{\text{intx}}$ and $k_{\text{force}}^{\text{intx}}$, we combined the mean-square discrepancies between the predicted and targeted $F_{\max}$ and ${\left[{\text{Ca}}^{2+}\right]}_{50}$ values into a cost function, mapped out in Fig. 3 *d*. The solid green contour represents the region where the test and Land models are consistent to within tolerances of $\sigma_{F\max}=3.0\;\text{kPa}$ and $\sigma_{{\text{Ca}}_{50}^{2+}}=0.2\;\mu M$ chosen to reflect typical experimental uncertainties. The green F-pCa curves in Fig. 3 *e*, obtained by sampling over this region, confirm the agreement with the Land model, represented by the black dashed curves, within the tolerance range.

We conclude that the steady-state predictions of the original Land model under isometric conditions are satisfactorily reproduced when the ad hoc length dependences in that model are replaced by an OFF-state dynamic model with feedback on the total force.

### Alternative feedback scenarios

**Figure 4:**
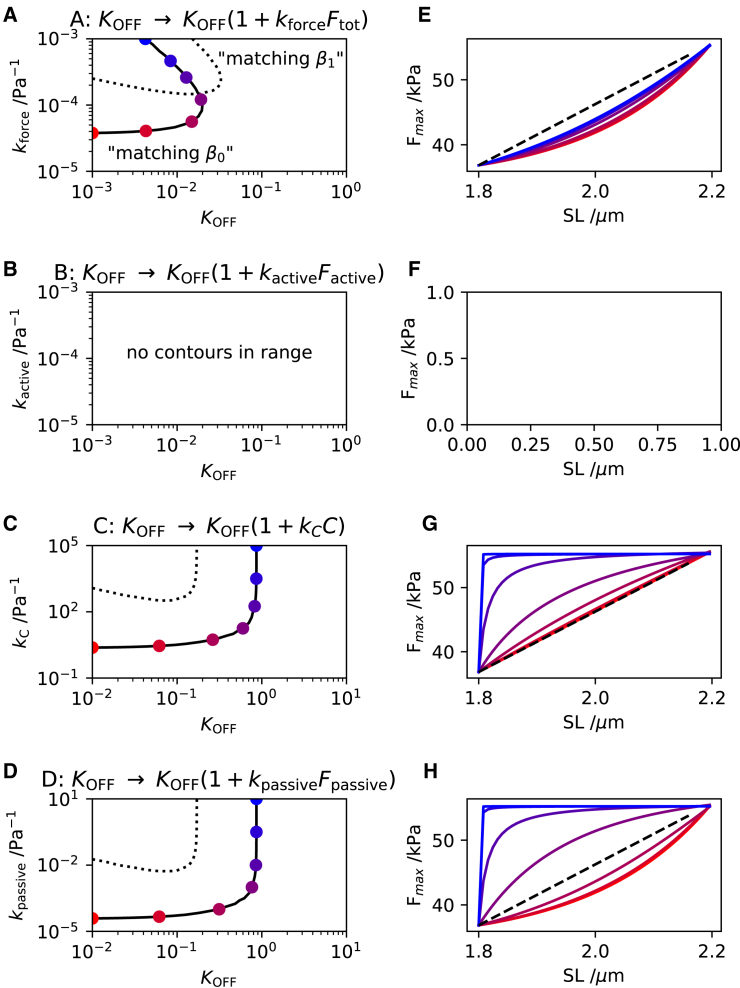
Exploring alternative feedback mechanisms. Contours representing the matching conditions $\beta_{0}^{\text{effective}}/\beta_{0}^{\text{Land}}=1$ (*solid curves*) and $\beta_{1}^{\text{effective}}/\beta_{1}^{\text{Land}}=1$ (*dotted curves*) are plotted, based on the different hypothetical feedback scenarios: (*a*) total force, (*b*) active force only, (*c*) passive force only, and (*d*) sarcomere strain $C=\text{SL}/{\text{SL}}_{0}-1$ (Eqs. 8, 9, and 10). Plots (*e*)–(*h*) show the length dependences of $F_{\max}$ corresponding to the colored markers in (*a*)–(*d*), respectively. To see this figure in color, go online.

The above analysis was repeated for paradigms B, C, and D to determine whether, with those feedback paradigms also, a similarly acceptable quality of match with the original Land model could be achieved. Fig. 4, *a–d* summarizes the outcome of these analyses by plotting, in each case and in the manner of Fig. 3 *d*, the corresponding contours of “matching $\beta_{0}$” and “matching $\beta_{1}$” (*left-hand side panels*). Overall, the plots suggest that only paradigm A (recapitulated in Fig. 4 *a*) displays intersecting contours, signifying that only this paradigm is capable of achieving simultaneously the required length dependences of $F_{\max}$ and ${\text{pCa}}_{50}$. An inspection of these graphical results suggests that only paradigm A allows the possibility of a contour intersection. Paradigms C and D show no sign of the contours converging; for paradigm B, the unit contours are conspicuously absent, signifying that no matching of either $\beta_{0}$ or $\beta_{1}$ was achieved within the parameter space considered.

The similarity in the outcomes for paradigms C and D (where the feedback is based, respectively, on strain and on passive force, Fig. 4, *c* and *d*) stems from the close relation between sarcomere strain and the passive tension (see Eq. 1). Indeed, these contours would be identical if the passive tension were a linear function of strain, i.e., with b≈0 in Eq. 1; trivially, the feedback gains $k_{\text{passive}}$ and $k_{C}$ would then be scaled values of one another. The absence of contour intersections in Fig. 4, *c* and *d* implies that the length dependences of $F_{\max}$ ($\beta_{0}$) and ${\text{pCa}}_{50}$ ($\beta_{1}$) in the original Land model could potentially be reproduced separately but not simultaneously by the amended models. The inadequacy of these models is further reinforced by considering the length dependence predictions at intermediate SL. The right-hand-side panels of Fig. 4 plot the length dependences of $F_{\max}$ corresponding to different locations along the contour shown in the left panels. All the curves intersect at SL = 1.8 and 2.2 *μ*m but they differ significantly in their degree of linearity at intermediate values. In the cases of Fig. 4, *g* and *h*, only a limited range of points along the contours provide a reasonable agreement with the linearity of the Land model (*dashed lines*) and as observed experimentally (37). In contrast, paradigm A achieves a better linearity generally, with the degree of linearity increasing further with increasing $k_{\text{force}}$. We therefore henceforth discard paradigms C and D as implausible paradigms.

With regard to paradigm B (where the feedback is based on active tension only, Fig. 4 *b*), no unit-value contours (“matching $\beta_{0}$” and “matching $\beta_{1}$”) are observed at all within the explored ranges of $K_{\text{OFF}}^{0}$ and $k_{\text{active}}$. This suggests that, for this case, the amended model was unable to reproduce the variations in $F_{\max}$ and ${\text{pCa}}_{50}$ displayed by the original Land model. This result is surprising at first sight since, at least in the regime of high $\left[{\text{Ca}}^{2+}\right]$, the total tension in the sarcomere is likely to be dominated by the active component. We note that paradigm B is, in effect, a variant of paradigm A in which the passive tension has been subtracted from the feedback mechanism. This points to the potential importance of the passive tension as a component of the OFF-state feedback mechanism. We therefore examined this effect, as described in the next section.

### Role of passive stiffness

**Figure 5:**
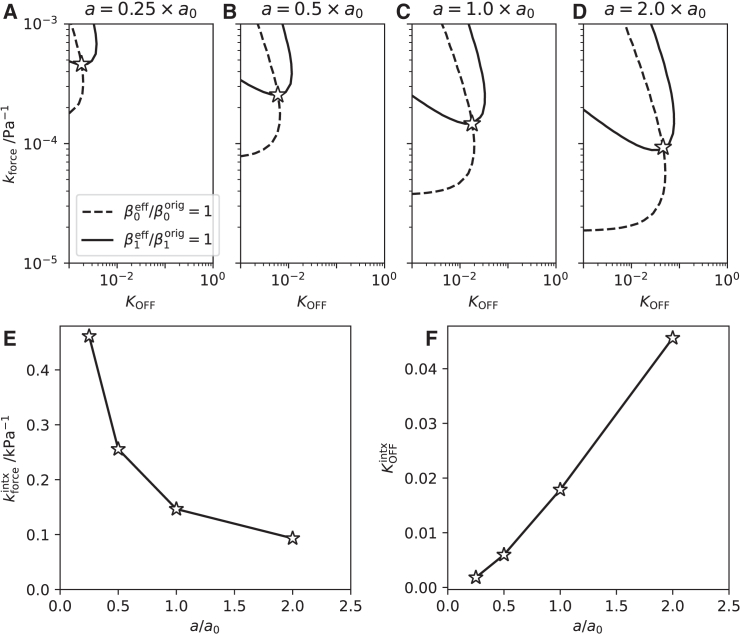
Effect of passive stiffness on force feedback. Contours representing $\beta_{0}^{\text{effective}}/\beta_{0}^{\text{Land}}=1$ and $\beta_{1}^{\text{effective}}/\beta_{1}^{\text{Land}}=1$ were calculated (see Fig. 3, *b* and *c*) for a range of values of the passive stiffness *a* (Eq. 1) equal to (*a*) 0.25×, (*b*) 0.5×, (*c*) 1.0×, and (*d*) $2.0\times a_{0}$, where $a_{0}=2.1\;\mathrm{kPa}$ is the reference stiffness value specified in the Land model (39). The coordinate values $k_{\text{force}}^{\text{intx}}$ and $K_{\text{OFF}}^{\text{intx}}$ of the intersection point are plotted in (*e*) and (*f*), respectively, as functions of $a/a_{0}$.

The analysis of Fig. 3 was repeated to determine, within paradigm A, the relevance of the stiffness parameter *a* (Eq. 1) on the $K_{\text{OFF}}^{0}$ and $k_{\text{force}}$ estimates. The original Land model specifies a stiffness $a_{0}=2.1\;\text{kPa}$. Fig. 5, *a*–*d* show the unit-value contours matching $\beta_{0}$ and $\beta_{1}$ for multiples of this reference value. As the stiffness decreases, both contours shift toward lower values of $K_{\text{OFF}}^{0}$ and higher values of $k_{\text{force}}$. Fig. 5, *e* and *f* plot the intersection coordinates $K_{\text{OFF}}^{\text{intx}}$ and $k_{\text{force}}^{\text{intx}}$ as functions of *a*. Specifically, $k_{\text{force}}^{\text{intx}}$ varies in inverse proportion to *a* while $K_{\text{OFF}}^{\text{intx}}$ approaches zero almost linearly. The divergence at zero stiffness suggests that, for this model, no intersection exists in the absence of a passive stiffness.

This result highlights the importance of the passive stiffness in enabling expected length dependences within the modeling framework of paradigm A. This paradigm assumes that length dependence arises entirely from the positive feedback effect in the OFF-state dynamics. As in any feedback system, this mechanism cannot achieve its function in the absence of some residual length-dependent force signal upon which to feed back. Having suppressed the intrinsic length dependences of the original Land model (embodied in the ad hoc parameters $\beta_{0}$ and $\beta_{1}$), the only remaining length-dependent contribution to force is in the passive stiffness (Eq. 1). This explains the absence of the unit contours in Fig. 4 *b* and, therefore, the inability of paradigm B to achieve the length dependences displayed by the Land model.

### Mavacamten

We tested the ability of our OFF-state modeling framework to predict experimentally measured responses to mavacamten, an OFF-state-stabilizing drug, as reported in the literature. A reduction in the magnitude of the generated force ($F_{\max}$) is generally observed, typically by an order of 30% for applied mavacamten concentrations of 0.5–1μM, consistent with a decrease in the number of available force-generating cross-bridges (44,45,49). We chose the experimental results of Awinda et al. (44), derived from human myocardium, as the main benchmark for testing the mavacamten model. Those measurements, performed at two sarcomere lengths 1.9 and 2.3 μm, include the F-pCa relationship and the frequency-dependent viscoelastic properties in the absence and presence of 0.5 μM mavacamten.

**Figure 6:**
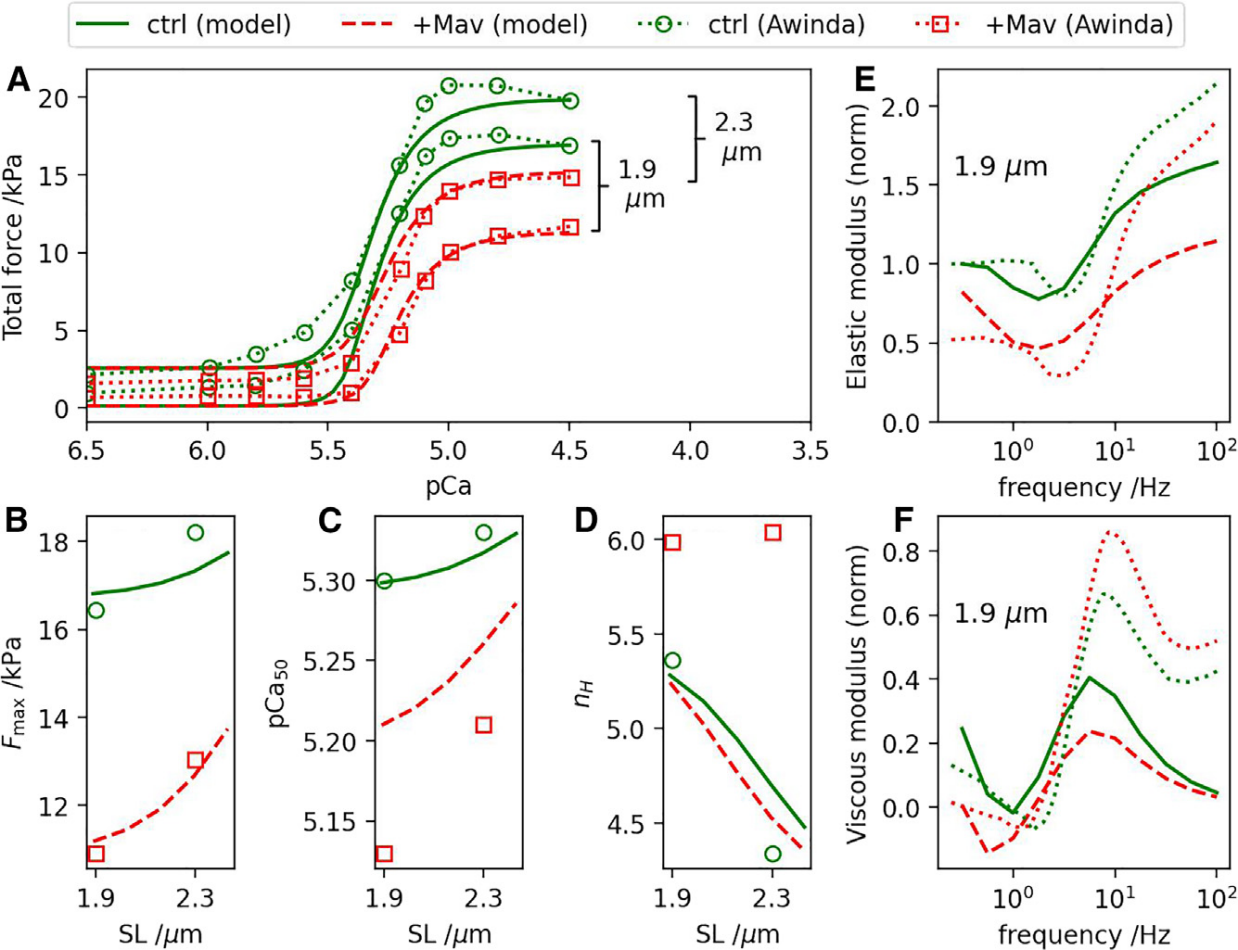
Comparing model simulations of mavacamten with the Awinda et al. measurements. (*a*) F-pCa relationships for SL = 1.9 and 2.3 μm as predicted by the recalibrated model simulations (*green solid curves*: “control—no mavacamten,” *red dashed curves*: “with mavacamten”), and copied from the Awinda et al. experimental measurements (44). The baseline model parameters differing from the original Land model are *a* = 0.241 kPa, *k* = 8.86, pCa50ref = 5.25, $k_{UW}$ = 4.98 s^−1^, $k_{WS}$ = 19.10 s^−1^, $\gamma_{S}$ = 42.1 s^−1^, $\gamma_{W}$ = 28.3 s^−1^, φ = 0.1498, $A_{\text{eff}}$ = 125, $\beta_{0}=\beta_{1}$ = 0, $T_{\text{ref}}$ = 23.0 kPa, $k_{1}$ = 0.877 s^−1^, $k_{2}$ = 12.6 s^−1^, $k_{\text{force}}$ = 1.44 kPa^−1^. Mavacamten was implemented by setting r=0.41 in Eq. 14. Corresponding Hill-curve features are plotted as functions of the sarcomere length: (*b*) $F_{\max}$, (*c*) ${\text{pCa}}_{50}$, and (*d*) $n_{H}$. Model-predicted (*green solid* and *red dashed curves*) and experimentally measured (44) (*dotted*) dynamic stiffness components are plotted, with and without mavacamten: (*e*) elastic and (*f*) viscous moduli. To see this figure in color, go online.

The model was first recalibrated to approximate the Awinda et al. control measurements (i.e., the combination of F-pCa and dynamic stiffness results, without mavacamten). The predictions of this recalibrated model are plotted in Fig. 6, *a–f*, *green solid curves*) alongside the Awinda et al. results (*green dotted curves* and *symbols*), showing general qualitative agreement. In particular, the modeled F-pCa curves display comparable SL dependences (increases in both $F_{\max}$ and ${\text{pCa}}_{50}$); the elastic modulus curves downward initially, reaching a minimum around 2–4Hz before increasing monotonically; the viscous modulus decreases at low frequency, dipping below zero around 1–2Hz and reaching a maximum around 10 Hz. It is noteworthy that the model predicts a negative SL dependence in $n_{H}$ that is not dissimilar to the measurements. The original Land model did not assign an ad hoc SL dependence to the cooperativity-related parameters (e.g., associated with troponin and tropomyosin), and is therefore incapable of producing this effect. (However, the Awinda control measurements contrast with other studies that show no correlation or a positive correlation (45).)

We applied mavacamten by setting r=0.41 in Eq. 14, a value that produced a ∼30% drop in $F_{\max}$ for SL=1.9μm as in the Awinda et al. measurements (Fig. 6, *a* and *b*, *red dashed curves*). As summarized in Table 1, most of the measured mavacamten response features are reproduced qualitatively by the simulations, in particular: the monotonic increasing of both $F_{\max}$ and ${\text{pCa}}_{50}$, the signature features of LDA, are preserved albeit with lower absolute values (Fig. 6, *b* and *c*); the gradient of ${\text{pCa}}_{50}$ versus SL is enhanced by mavacamten (Fig. 6 *c*); the elastic modulus is reduced over the entire range of frequencies while maintaining the same overall functional form (Fig. 6 *e*); the positive values of the viscous modulus at the low end of the frequency range are suppressed to near zero upon application of mavacamten; the frequency of the viscous modulus minimum is decreased (Fig. 6 *f*: Δf∼−0.3Hz in the simulations, ∼−0.7Hz in the measurements); and the frequency of viscous modulus maximum is increased (Δf∼0.9Hz in the simulations, ∼1.1Hz in the measurements).

**Table 1:** Comparison summary between the mavacamten model predictions and experimental observations

Awinda et al. 2020 (human myocardium) (44) experimental observations	Simulation results
Mav reduces $F_{\max}$ by ∼30% at short SL	✓
Mav preserves the ability of stretch to increase $F_{\max}$	✓
Mav preserves the ability of stretch to increase ${\text{pCa}}_{50}$	✓
Mav reduces ${\text{pCa}}_{50}$ at both short and long SL	✓
Mav enhances gradient of ${\text{pCa}}_{50}$ versus SL	✓
Mav suppresses SL dependence of $n_{H}$	×
Mav reduces elastic modulus (high frequency)	✓
Mav reduces elastic modulus (low frequency)	✓
Mav reduces magnitude of negative viscous modulus	×
Mav reduces frequency of viscous modulus minimum	✓
Mav increases frequency of viscous modulus maximum	✓

Ma et al. 2021 (pig myocardium) (45) experimental observations	Simulation results
Mav reduces $F_{\max}$ by ∼32% at short SL	✓
Mav preserves the ability of stretch to increase $F_{\max}$	✓
Mav does not significantly affect relative change in $F_{\max}$ with SL	✓
Mav does not affect relative change in $n_{H}$ with SL	(✓)
Mav does not affect ${\text{pCa}}_{50}$ at short SL	×
Mav does not affect $n_{H}$ at short SL	✓
Mav abolishes SL dependence of ${\text{pCa}}_{50}$	×

Experimental results are by Awinda et al. (human myocardium) (44) and Ma et al. (pig myocardium) (45). The OFF-state model was calibrated so as to achieve a satisfactory match with the control measurements (no mavacamten) of Awinda et al. (see Fig. 6 caption). Tick marks ✓ denote qualitative agreement with the experimental observations, whereas × denotes disagreement. The tick mark in brackets indicates agreement with a caveat (see text).

The mavacamten simulations nonetheless display several discrepancies with the Awinda et al. data. Firstly, in Fig. 6 *d*, whereas the measurements show a suppression of the SL dependence of $n_{H}$ by mavacamten, the impact of mavacamten on this simulation result is negligible. This may be explained by the original Land model having a length-independent Hill coefficient, consistent with the Dobesh measurements it was based on (6). Secondly, in Fig. 6 *f*, whereas Awinda et al. observe a small shift in the minimum viscous modulus toward less negative value, the simulation results display increased negative values.

We also compared the mavacamten simulations with the experimental findings of Ma et al. derived from pig myocardium. The comparison is also summarized in Table 1. Although some of the Ma et al. results are consistent with those of Awinda et al., there are notable differences that may reflect species-specific differences in the muscle system or perhaps details of the measurement method. Both studies report a comparable drop in $F_{\max}$ upon addition of mavacamten, as observed in our simulations. However, Ma et al. observe a total suppression of the length dependence of ${\text{pCa}}_{50}$ (45) in contrast with Awinda et al., where the rate of increase in ${\text{pCa}}_{50}$ with SL is instead enhanced by mavacamten (44). Whereas Awinda et al. report a suppression of the SL dependence of $n_{H}$ by mavacamten, Ma et al. observe no significant impact. This observation matches our simulation results (Fig. 6 *d*), with the caveat that Ma et al. observed an *increase* in $n_{H}$ with SL, while our simulations give the opposite result when calibrated to Awinda et al. control data.

In summary, the model predictions agree with the main characteristic features of LDA with regard to the preservation of the ability of stretch to vary both $F_{\max}$ and ${\text{pCa}}_{50}$, albeit with reduced absolute values of these quantities.

## Discussion

The aim of the present study was to test whether myosin OFF-state dynamics can in principle account for the observed length-dependent activation of contraction of the cardiac sarcomere. To achieve this, we adopted a graphical approach to assess the intrinsic ability of the model to satisfy multiple requirements simultaneously, namely with regard to the $F_{\max}$ and ${\text{pCa}}_{50}$ length dependences. A more direct numerical error-minimization approach, while capable of yielding a “best model,” would have been less suitable for this categorical assessment. Our main conclusion is that the inclusion of a force-dependent feedback mechanism for myosin-head recruitment from the OFF state to the ON state is indeed able to reproduce observed length dependences of force generation (force magnitude and ${\text{Ca}}^{2+}$ sensitivity) as expressed by the Land 2017 human left-ventricle model (39). Our results suggest that the OFF state is in principle capable of accounting for both these LDA characteristics unaided by other mechanisms. Specifically, these features of the Land model are precisely emulated by replacing the ad hoc LDA parameters $\beta_{0}$ and $\beta_{1}$ with an OFF-state subsystem, with dynamics characterized by $K_{\text{OFF}}^{0}=0.0158$ and $k_{\text{force}}=1.48\times 10^{-4}{\;\text{Pa}}^{-1}$. The low value of $K_{\text{OFF}}^{0}$ suggests that, in the absence of tension ($F_{\text{total}}=0$), myosin heads are predominantly in the OFF configuration. As the tension increases to an order of $F_{\text{total}}∼50\;\text{kPa}$, $K_{\text{OFF}}$ increases by up to two orders of magnitude ($K_{\text{OFF}}=K_{\text{OFF}}^{0}\times \left[1+k_{\text{force}}F_{\text{total}}\right]∼1.3$), corresponding to the OFF and ON state populations becoming comparable. As we demonstrate, LDA behavior emerges naturally from this OFF-state dynamics.

The plausibility of the proposed mechanism does not preclude other contributions to LDA. Alternative mechanistic explanations of length dependence, unrelated to the OFF state, may well play a significant role, e.g., involving interfilament interactions (9,10,18), thin-filament cooperativity (14,15), TnC affinity (11,12,13), MyBP-C (16,17), or a force dependence of the detachment rate and duty ratio (50,51). They can in principle be accommodated within the present framework in a hybrid fashion, using intermediate values of $\beta_{0}$ and $\beta_{1}$ alongside the OFF-state dynamics. The exact balance of these contributions, however, would remain to be established. This notwithstanding, the present study highlights the potentially predominant role of the OFF state in accounting for LDA and the Frank-Starling effect.

Campbell et al. previously incorporated a tension-dependent (with total tension = active + passive) OFF-to-ON rate constant in a kinetic cross-bridge model (40,41). Simulations reproduced observed behavior better when including force dependence than without including it, in particular with regard to ${\text{pCa}}_{50}$ and $F_{\max}$. The present work sought to formalize this choice of formulation and shows that a simpler distortion-decay formulation of sarcomere dynamics can formally capture the essential underlying mechanosensing effect and predict its functional impact.

Computational muscle contraction modeling has been enriched over the years by various styles of analysis, involving partial differential equations (52,53,54) and ODEs (37,55,56,57,58), as well as spatially explicit models (59,60,61). These approaches contribute complementary insights, balancing molecular detail with computational efficiency and parameter-set complexity. Although mass-action-based ODE models inevitably neglect detailed structural configurations that may influence contraction, their more phenomenological nature may also safeguard against attributing observable effects to inadequately or incompletely characterized specific mechanisms. Their relative simplicity arguably limits the scope for ambiguous or arbitrary calibration and, ultimately, facilitates their incorporation into multiscale whole-organ simulations (47).

This appeal was part of the motivation of our chosen modeling approach. The results are relevant for understanding fundamental mechanisms of contraction, particularly in the context of pharmacological treatments with drugs such as mavacamten that target the OFF state. Our proposed modeling framework accommodates the simulation of mavacamten, reproducing qualitatively many of the essential features of the drug observed experimentally in particular by Awinda et al. (44) and Ma et al. (45) in relation to the $F_{\max}$ and ${\text{pCa}}_{50}$ length dependences.

The mavacamten simulations differ with respect to the Hill coefficient. Three reasons can explain this. First, the Hill coefficient in the original Land model, informed by the measurement of Dobesh et al., had no length dependence. Second, there are inconsistent experimental reports of the effects of mavacamten on the Hill coefficient, with Awinda et al. reporting a decrease in length-dependent Hill coefficient while Ma reports no change. Third, the effects of OFF-state dynamics not included in the present model (e.g., the explicit formulation of cross-bridge binding rates) may be significant. It is not possible currently to make a model that replicates all of the observed effects of mavacamten on the length dependence of the Hill coefficient.

Another discrepancy between the simulation and the measurements appears in the change in magnitude of the negative viscous moduli induced by mavacamten at low frequencies (<2Hz). Whereas the measurements display a shift in the minimum viscous modulus toward less negative values, the simulations predict instead a significant enhancement of the minimum toward more negative values. Awinda et al. interpret the observed suppression in terms of a reduction in the net oscillatory work produced upon application of mavacamten (44,62). This interpretation is intuitively consistent with the expected reduction in the number of cross-bridges available for producing work. Our proposed mavacamten model does not produce this effect while inherently reducing the proportion of available cross-bridges. We regard this as a limitation of our relatively simple model of mavacamten action that would require further development to capture these secondary effects displayed by complex experiments.

The proposed model structure suggests a natural role for the passive tension, insofar as it provides the initial length-dependent signal upon which to feed back. This outcome is formally inevitable if we consider the explicit solutions of the model equations (see appendix), where the source of the length dependence resides in $F_{\text{passive}}$ before subsequent magnification via $F_{\text{total}}$. This passive tension was assumed to result from the inherent length dependence of an elastic component, attributed to titin. The relevance of passive tension in regulating muscle contraction is becoming increasingly recognized but the precise mechanism remains to be clarified (26,28,63). This role may vary according to muscle type. Fusi et al. observed thick-filament activation (via changes in the myosin regulatory light-chain orientation in skeletal muscle, imaged using polarized fluorescence) even under low-$\left[{\text{Ca}}^{2+}\right]$ conditions (pCa 9.0), where active tension is suppressed (24). Ait-Mou et al. observed LDA in rat myocardium experiments being dependent on the titin isoform, suggesting also a dependence on passive stress (28). In contrast, Reconditi et al. reported the thick filaments being in the OFF configuration regardless of the diastolic sarcomere length, and therefore of passive tension (30); the proportion of ON myosins then depends on *systolic* tension, i.e., the active force during twitch. Similarly, Park-Holohan et al., also using rat trabeculae, reported an overwhelmingly stronger dependence of the OFF/ON ratio on active than passive tension, and therefore dismissed the hypothesis that LDA (and hence the Frank-Starling effect) is triggered by passive stress (64). By including titin-based passive tension in a mathematical model (Monte Carlo simulation), Marcucci et al. (65) reproduced qualitatively a length dependence of $\left[{\text{Ca}}^{2+}\right]$ sensitivity (also by avoiding ad hoc assumptions of length dependence) but not of $F_{\max}$. In a subsequent development of that model, the effect on $F_{\max}$ was also reproduced by invoking a nonuniformity of tension over the thick filament, the extent of which varies with length (66). We believe that this interpretation is not necessarily incompatible with our ODE representation of the filament interactions, which of course does not explicitly resolve the behavior of individual cross-bridges.

Early suggestions of the existence of intrinsic myosin regulation were based on the observation of strongly dissimilar ATPase rates between a so-called disordered relaxed (DRX) state and super-relaxed (SRX) state (67). Such biphasic ATPase behavior requires the coexistence of the two distinct myosin states far from equilibrium (68), a condition unlikely to be met by the ON and OFF states of our model. However, recent studies have questioned the often-assumed equivalence of DRX/SRX with ON/OFF, despite their possible correlation under some conditions (69,70). As our basic understanding of thick-filament regulation progresses, the correct interpretation of the different experiments remains the subject of ongoing debate.

Other recent studies have proposed alternative modeling implementations of mavacamten. Margara et al. recently simulated the impact of mavacamten, also using an adaptation of the original Land 2017 model (39), by phenomenologically suppressing the proportion of available myosin heads (71). This was achieved practically by modulating the transition rates of binding and unbinding to actin (i.e., the transition from state *U* to *W*), taken as functions of the mavacamten dose. The effect on thin-filament activation was also included by reformulating the calcium sensitivity as a function of the myosin availability, recalibrated by an empirical fit. However, this pragmatic approach comes at the expense of introducing further phenomenological components to the model, characterized by yet more ad hoc parameters.

## Conclusion

The present modeling framework provides a minimal ODE description of the OFF-state dynamics capable of reproducing the essential functional characteristics of thick-filament activation, arising as the consequence of a specific biophysical mechanism. Compared with more sophisticated spatially explicit sarcomere models, ODE-based models such as the Land 2017 model generally benefit from having a lower dimensionality and a lower computational cost, making them amenable to a more convenient integration within multiscale computational models. This in turn may benefit studies through the computational modeling of whole-heart contraction, of the mode of action and clinical applications of pharmacological treatments of cardiac pathologies (46,47). Such treatments may either impede (e.g., mavacamten) or favor the release of OFF myosins to ON (e.g., danicamtiv (72,73,74) or 2-deoxy-ATP (75,76)). The proposed modeling framework could be adapted and calibrated to describe these treatments.

## Author contributions

A.L. designed the research, performed the simulations, analyzed the results, and wrote the manuscript. G.M. and K.S.C. assisted in the results analysis and discussion and edited the manuscript. S.A.N. assisted in the research design, the results analysis, and writing the manuscript.

## Appendix

### ODE system

The present model inherits the ODEs of the original Land model (39), amended to include additional terms representing the transitions into and out of the new OFF states $B_{\text{off}}$ and $U_{\text{off}}$ in Fig. 1:

$$\frac{dB}{dt}=k_{b}\cdot {\text{CaTRPN}}^{-n_{\text{Tm}}/2}\cdot U-k_{u}\cdot {\text{CaTRPN}}^{n_{\text{Tm}}/2}\cdot B+k_{1}\cdot B_{\text{off}}-k_{2}\cdot B$$

$$\frac{dB_{\text{off}}}{dt}=k_{b}\cdot {\text{CaTRPN}}^{-n_{\text{Tm}}/2}\cdot U_{\text{off}}-k_{u}\cdot {\text{CaTRPN}}^{+n_{\text{Tm}}/2}\cdot B_{\text{off}}+k_{2}\cdot B-k_{1}\cdot B_{\text{off}}$$

$$\frac{dU_{\text{off}}}{dt}=-k_{b}\cdot {\text{CaTRPN}}^{-n_{\text{Tm}}/2}\cdot U_{\text{off}}+k_{u}\cdot {\text{CaTRPN}}^{+n_{\text{Tm}}/2}\cdot B_{\text{off}}+k_{2}\cdot U-k_{1}\cdot U_{\text{off}}$$

$$\frac{dW}{dt}=k_{UW}U-k_{SU}W-k_{WS}W-\gamma_{WU}\left(\zeta_{W}\right)W$$

$$\frac{dS}{dt}=k_{WS}W-k_{SU}S-\gamma_{SU}\left(\zeta_{S}\right)S$$

(Equation 15) $$U=1-B-W-S-B_{\text{off}}-U_{\text{off}}$$

where CaTRPN is the proportion of calcium-bound troponin C units

$$d\text{CaTRPN}/dt=k_{\text{TRPN}}\left[{\left(\left[{\text{Ca}}^{2+}\right]/{\left[{\text{Ca}}^{2+}\right]}_{50}^{\text{Trpn},\text{ref}}\right)}^{n_{\text{TRPN}}}\left(1-\text{CaTRPN}\right)-\text{CaTRPN}\right]\text{,}$$

and $k_{b}$, $k_{u}$, $k_{UW}$, $k_{WU}$, $k_{WS}$, $k_{SU}$, and $k_{\text{TRPN}}$ are transition rate constants. $\gamma_{\text{WU}}\left(\zeta_{W}\right)$ and $\gamma_{\text{SU}}\left(\zeta_{S}\right)$ represent cross-bridge unbinding rate constants for the *W* and *S* states, and are assumed to depend on the cross-bridge distortions $\zeta_{W}$ and $\zeta_{S}$. These distortions are treated as state variables (in addition to the state populations), obeying

$$d\zeta_{W}/dt=A\;d\lambda /dt-c_{W}\;\zeta_{W}$$

(Equation 16) $$d\zeta_{S}/dt=A\;d\lambda /dt-c_{S}\;\zeta_{S}$$

where $\lambda =\text{SL}/{\text{SL}}_{0}$ is the relative cell length relative to the resting length, *A* is a constant relating the change in sarcomere length to cross-bridge distortion, and the distortion decay rate constants $c_{W}$ and $c_{S}$ are assumed to be proportional to the steady-state cross-bridge cycling rates under isometric conditions:

$$c_{W}=\varphi \cdot U_{\text{ss}}/S_{\text{ss}}$$

$$c_{S}=\varphi \cdot S_{\text{ss}}/S_{\text{ss}}$$

where φ is a constant. The cross-bridge detachment rate constants (in dW/dt and dS/dt above) are then given by

$$\gamma_{WU}\left(\zeta_{W}\right)=\gamma_{W}\left|\zeta_{W}\right|$$

(Equation 17) $$\gamma_{SU}\left(\zeta_{S}\right)=\left\{\begin{array}{cc} -\gamma_{S}\left(1+\zeta_{S}\right) & \zeta_{S}<-1\\ 0 & \zeta_{S}\in \left[-1,0\right]\\ \gamma_{S}\zeta_{S} & \zeta_{S}>0 \end{array}\right)$$

### Closed-form solution in the isometric steady state

The isometric steady state of the model is determined by equating the left hand side of each ODE to zero for constant λ. The relative populations of the states are given by the equilibrium constants:

$$K_{\text{ub}}=\frac{U_{\text{ss}}}{B_{\text{ss}}}=\frac{U_{\text{off},\text{ss}}}{B_{\text{off},\text{ss}}}=\frac{k_{u}}{k_{b}}{\text{CaTRPN}}_{\text{ss}}^{+n_{\text{Tm}}}$$

$$K_{\text{OFF}}=\frac{U_{\text{ss}}}{U_{\text{off},\text{ss}}}=\frac{B_{\text{ss}}}{B_{\text{off},\text{ss}}}=\frac{k_{1}}{k_{2}}$$

In the isometric steady state, the active force (Equation 53 in (39)) reduces to

$$F_{\text{active},\text{ss}}=h\left(\lambda \right)\frac{T_{\text{ref}}}{r_{S}}S_{\text{ss}}$$

(Equation 18) $$=h\left(\lambda \right)\underset{\mu }{\underset{︸}{\frac{T_{\text{ref}}}{r_{S}}\cdot \frac{k_{WS}k_{UW}}{k_{SU}\left(k_{SU}+k_{WS}\right)}}}U_{\text{ss}}$$

The amended OFF-state model replaces the ad hoc length dependence of h(λ) with the OFF-state dynamics to the extent that $B_{\text{off}}$ and $U_{\text{off}}$ (which are effectively length dependent) depopulate states *U* and *S*. The OFF states do not, however, alter the proportionality relation between $U_{\text{ss}}$ and $S_{\text{ss}}$ given in Eq. 18.

Assuming feedback paradigm A (see main text), in which the OFF-state dynamics is a function of the total force, we have

(Equation 19) $$K_{\text{OFF}}=K_{\text{OFF}}^{0}\times \left[1+k_{\text{force}}\left(F_{\text{active},\text{ss}}+F_{\text{passive}}\right)\right]$$

Applying the conservation constraint $B_{\text{ss}}+B_{\text{off},\text{ss}}+U_{\text{ss}}+U_{\text{off},\text{ss}}+S_{\text{ss}}+S_{\text{ss}}=1$ and substituting the equilibrium conditions derived above, we obtain

(Equation 20) $$U_{\text{ss}}={\left[\left(1+\frac{1}{K_{ub}}\right)\left(1+\frac{1}{K_{\text{OFF}}}\right)+Q\right]}^{-1}$$

where

$$Q=\frac{k_{WS}k_{UW}}{k_{SU}\left(k_{SU}+k_{WS}\right)}+\frac{k_{UW}}{k_{SU}+k_{WS}}\text{.}$$

$K_{\text{OFF}}$ in Eq. 20 implicitly contains the force dependence shown in Eq. 19 and, consequently, $U_{\text{ss}}$ via Eq. 18. Substituting Eqs. 18 and 19 into Eq. 20, we obtain a quadratic equation for $U_{\text{ss}}$, giving the solution

(Equation 21) $$U_{\text{ss}}=\frac{\beta \left[-1+{\left(1+\frac{4\mu \left(1+\frac{1}{K_{\text{ub}}}+Q\right)\left(1+k_{\text{force}}F_{\text{passive}}\right)}{\beta^{2}}\right)}^{1/2}\right]}{2\mu \left(1+\frac{1}{K_{\text{ub}}}+Q\right)}$$

where

$$\beta =\left(1+k_{\text{force}}F_{\text{passive}}\right)\left(1+\frac{1}{K_{\text{ub}}}+Q\right)+\frac{1}{K_{\text{OFF}}^{0}}\left(1+\frac{1}{K_{\text{ub}}}\right)-\mu$$

and μ is the constant prefactor defined in Eq. 18. Under isometric conditions, the steady-state solution for the total force, $F_{\text{total},\text{ss}}=F_{\text{active},\text{ss}}+F_{\text{passive},\text{ss}}$ is hence obtained by substituting Eq. 21 into Eq. 18. This solution is implicitly $\left[{\text{Ca}}^{2+}\right]$ dependent via the state variable CaTRPN (see (39) and SL dependent via $F_{\text{passive},\text{ss}}$).

### Computer code

Computer code for the model implementation can be found at https://github.com/AlexLewalle/OFFstateFfeedback.
